# Cholera Toxin Induces Sustained Hyperexcitability in Myenteric, but Not Submucosal, AH Neurons in Guinea Pig Jejunum

**DOI:** 10.3389/fphys.2017.00254

**Published:** 2017-04-27

**Authors:** Katerina Koussoulas, Rachel M. Gwynne, Jaime P. P. Foong, Joel C. Bornstein

**Affiliations:** Enteric Neuroscience Laboratory, Department of Physiology, University of MelbourneParkville, VIC, Australia

**Keywords:** neurokinin 1 (NK1) antagonist, NK3 antagonist, after-hyperpolarization (AH), myenteric plexus (MP), submucosal plexus (SMP)

## Abstract

**Background and Aims:** Cholera toxin (CT)-induced hypersecretion requires activation of secretomotor pathways in the enteric nervous system (ENS). AH neurons, which have been identified as a population of intrinsic sensory neurons (ISNs), are a source of excitatory input to the secretomotor pathways. We therefore examined effects of CT in the intestinal lumen on myenteric and submucosal AH neurons.

**Methods:** Isolated segments of guinea pig jejunum were incubated for 90 min with saline plus CT (12.5 μg/ml) or CT + neurotransmitter antagonist, or CT + tetrodotoxin (TTX) in their lumen. After washing CT away, submucosal or myenteric plexus preparations were dissected keeping circumferentially adjacent mucosa intact. Submucosal AH neurons were impaled adjacent to intact mucosa and myenteric AH neurons were impaled adjacent to, more than 5 mm from, and in the absence of intact mucosa. Neuronal excitability was monitored by injecting 500 ms current pulses through the recording electrode.

**Results:** After CT pre-treatment, excitability of myenteric AH neurons adjacent to intact mucosa (*n* = 29) was greater than that of control neurons (*n* = 24), but submucosal AH neurons (*n* = 33, control *n* = 27) were unaffected. CT also induced excitability increases in myenteric AH neurons impaled distant from the mucosa (*n* = 6) or in its absence (*n* = 5). Coincubation with tetrodotoxin or SR142801 (NK3 receptor antagonist), but not SR140333 (NK1 antagonist) or granisetron (5-HT_3_ receptor antagonist) prevented the increased excitability induced by CT. Increased excitability was associated with a reduction in the characteristic AHP and an increase in the ADP of these neurons, but not a change in the hyperpolarization-activated inward current, *I*_*h*_.

**Conclusions:** CT increases excitability of myenteric, but not submucosal, AH neurons. This is neurally mediated and depends on NK3, but not 5-HT_3_ receptors. Therefore, CT may act to amplify the secretomotor response to CT via an increase in the activity of the afferent limb of the enteric reflex circuitry.

## Introduction

When the bacterium *Vibrio cholera* invades the gut, its exotoxin, cholera toxin (CT), causes hypersecretion in the small intestine, which can produce severe diarrhea that quickly leads to dehydration and death if left untreated. The enteric nervous system (ENS) has been implicated in the harmful effects of CT in the small intestine since the 1980s (Cassuto et al., 1981, 1982). The ENS is a complex nerve circuitry embedded within the walls of the gastrointestinal tract which regulates vital gut functions, including motility and secretion. It incorporates two distinct ganglionated networks- the myenteric (MP) and submucosal (SMP) plexuses.

In one long-standing model of toxin-induced hypersecretion, CT is postulated to activate persistent release of 5-HT from the mucosa, which then activates secretomotor reflex pathways in the ENS. The components of the neuronal reflex pathways include 5-HT_3_ receptors and probably several neuronal subtypes from each plexus communicating via nicotinic synapses. The secretomotor efferents release vasoactive intestinal peptide (VIP) which binds to specific receptors on enterocytes, activating an adenylyl cyclase-cAMP pathway to drive water and electrolyte secretion (Farthing, 2000; Lundgren, 2002). However, effects of CT are more complex than this. Recent work has focused on the effects of CT exposure on the properties of the enteric neurons in the secretomotor and motility reflex pathways (Kordasti et al., 2006; Gwynne et al., 2009; Fung et al., 2010). We have previously shown that luminal incubation of CT in isolated guinea pig jejunum induces a sustained increase in excitability of submucosal secretomotor neurons, the final neurons of the secretomotor pathways (Gwynne et al., 2009). Thus, one action of CT is to enhance the response of secretomotor neurons to activity in secretomotor pathways, but the question remains as to whether the properties of other elements of these pathways are also altered.

Intrinsic sensory neurons (ISNs) are central to the enteric circuitry and are the initial neurons in the secretomotor pathways. They are also referred to as intrinsic primary afferent neurons (IPANS) (Kirchgessner and Gershon, 1988) or as AH-type neurons from their distinctive electrophysiological feature of a prolonged after-hyperpolarizing potential (AHP) following an action potential. In this paper, we refer to them as AH neurons. The prolonged AHP is critical in determining the excitability of AH neurons, since it has the capacity to limit firing rate and slow excitatory transmission (Bertrand and Thomas, 2004). Other currents exhibited by AH neurons are also important for the regulation of cell excitability (Galligan et al., 1990; Rugiero et al., 2002; Nguyen et al., 2005; Chambers et al., 2014). There are also mechanosensitive myenteric neurons in guinea pig small intestine that lack the characteristics of AH neurons (for review see Schemann and Mazzuoli, 2010); in guinea pig colon, similar neurons appear to be S-type interneurons (Spencer and Smith, 2004), Whether they have sensory functions in small intestinal reflex pathways remains to be established.

AH neurons are present in the SMP and MP (Furness, 2000). In the MP, AH neurons project circumferentially and synapse with other AH neurons, as well as virtually all other types of neurons (Bornstein et al., 1991b; Kunze et al., 1993; Pompolo and Furness, 1998) and this may also be the case in the SMP (Song et al., 1992; Evans et al., 1994; Pan and Gershon, 2000; Reed and Vanner, 2001). Thus, AH neurons form interconnected networks (Thomas et al., 2000; Bornstein et al., 2012). Given this ability to form networks, AH neurons are not always strictly “afferent,” as they can act as interneurons under some conditions (Wood, 1994; Bertrand et al., 1997; Kunze and Furness, 1999; Thomas and Bornstein, 2003; Chambers et al., 2005).

In this study we used intracellular recording to analyse the effects of pre-treatment with CT in the lumen on the firing of AH submucosal AH neurons and myenteric AH and S (the other major electrophysiological class of neuron in the myenteric plexus) in the guinea pig jejunum. We found that prior acute exposure to CT enhanced the firing of myenteric AH neurons, but not that of myenteric S neurons or submucosal AH neurons. This effect was neurally mediated and depended on activation of NK_3_ tachykinin receptors, but not 5-HT_3_ receptors.

## Methods

### Tissue preparation

Experiments were performed using guinea pigs (weighing 170–350 g) of either sex. The animals were killed by stunning and then having their carotid arteries and spinal cords severed, in accordance with the guidelines of the University of Melbourne Animal Experimentation Ethics Committee. The abdominal cavity was cut open and a 5–10 cm segment of jejunum immediately distal to the duodenal-jejunal junction was removed. The tissue was first flushed clean of any contents and then placed in a dissecting dish lined with silicone elastomer (Sylgard 184, Dow Corning, North Ryde, NSW, Australia) in physiological saline (composition in mM per liter: NaCl, 118; KCl, 4.6; CaCl_2_, 2.5; MgSO_4_, 1.2; NaH_2_PO_4_, 1; NaHCO_3_ 25; d-glucose, 11), bubbled with 95% O_2_, 5% CO_2_. The lumen of the jejunal segment was injected with 0.3–0.5 mL of either physiological saline (control), CT (12.5 μg/mL) in saline or CT with an antagonist and then tied off at each end, as described in Gwynne et al. (2009), and transferred into a beaker containing physiological saline equilibrated with 95% O_2_, 5% CO_2_ and kept at 35°C.

After a 90 min incubation, the lumen of the jejunal segment was flushed clear of its contents with physiological saline, with care being taken to avoid the contents coming into contact with the serosa. The tissue was then kept in physiological saline containing nicardipine (1.25 μM) and hyoscine (1 μM) to prevent smooth muscle contractions. The segment was cut open along the mesenteric border and pinned flat with the mucosal side up in a Sylgard (184, Dow Corning, North Ryde, NSW, Australia) -lined dissecting dish. Three different electrophysiological preparations were produced by microdissection: (1) mucosa-LMMP preparations, where the full thickness of the gut wall was left intact in one-half, while the myenteric plexus with attached longitudinal muscle (LMMP) was exposed in the other half (Kunze et al., 1995; Bertrand et al., 1998, 2000; Gwynne and Bornstein, 2009); (2) mucosa-SMP preparations, where the mucosa and submucosa layers were left intact in one-half, but the outer muscle layers including the MP were removed, while the mucosa was removed from the other half of the preparation exposing the SMP (Gwynne et al., 2009); and (3) conventional LMMP preparations, where the mucosa and submucosa were completely removed. Each preparation was transferred to a recording bath continually superfused with 35–36°C physiological saline bubbled with 95%O_2_/5%CO_2_ and pinned flat mucosal side up. The preparation was positioned so its mucosal half was closest to the bath outflow to minimize diffusion of substances sloughing off the mucosa into the recording area (exposed MP or SMP) (Gwynne et al., 2009). The preparation was then allowed to equilibrate for an hour before commencing electrophysiological experiments.

### Intracellular recording

Neurons were impaled with intracellular glass recording microelectrodes (95–200 MΩ tip resistance) containing 1 mol/L KCl, with or without 2% biocytin (Sigma Aldrich, Castle Hill, NSW, Australia) to allow them to be identified morphologically and immunohistochemically after completion of the electrophysiological recordings. Recordings were taken from neurons in ganglia in the “first row” close to the mucosal wall in both mucosa-LMMP and mucosa-SMP preparations. Recordings were also made up to 1 cm away from the edge of the mucosa in mucosa-LMMP preparations and from any ganglion in LMMP preparations lacking all mucosa.

**Figure 1:**
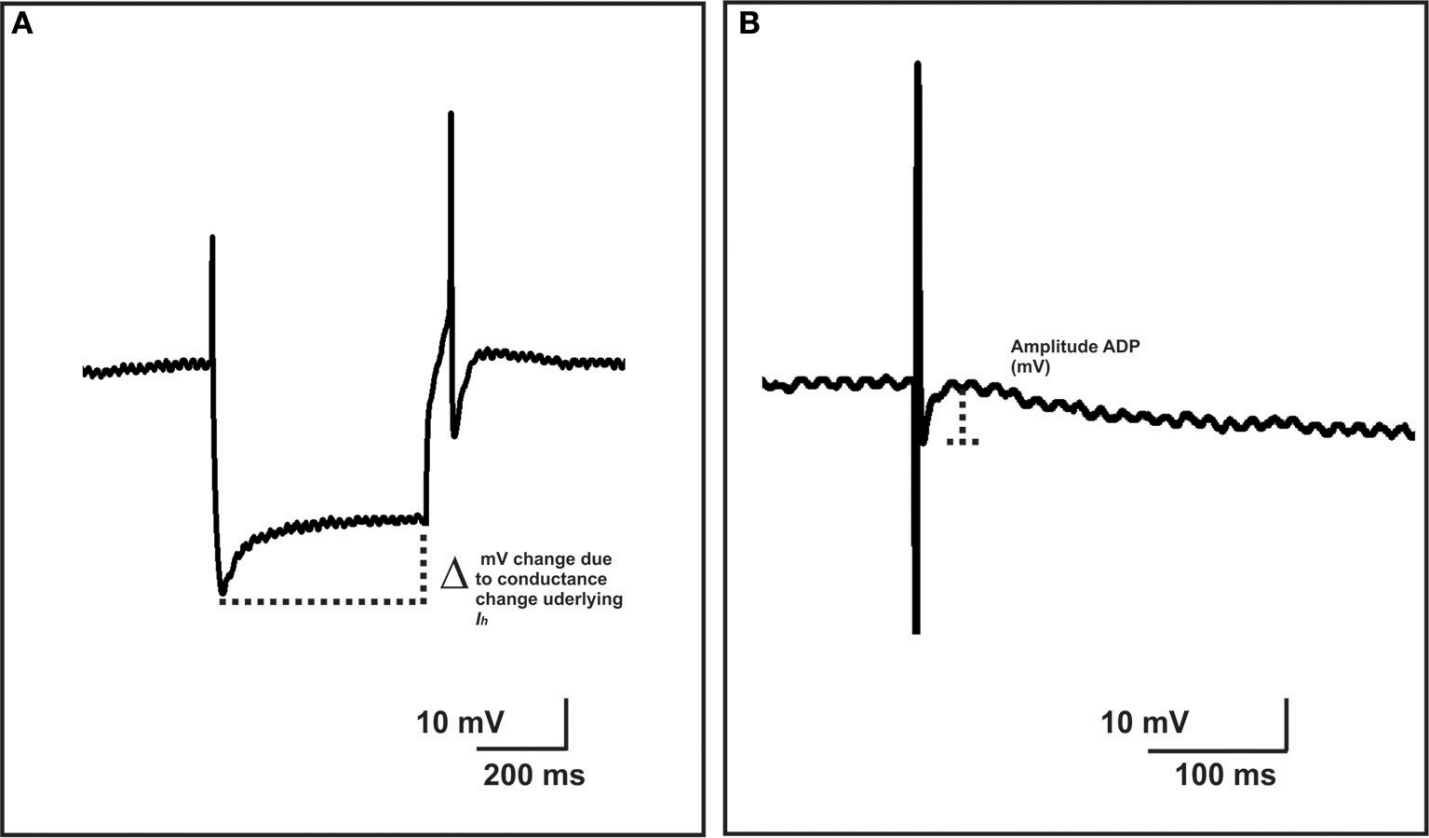
**Measurements of *I*_h_-induced rectification and ADP amplitude**. The magnitude of the *I*_h_-induced rectification, was measured as the difference between the maximum hyperpolarization and the hyperpolarization at the end of each current step **(A)**. The gradient of this voltage current relationship was measured in the same way as the input resistance of the cell (see Methods) and represents the change in input resistance due to the conductance change underlying the *I*_h_ current. The amplitude of an after-depolarizing potential (ADP) was measured from the base of the ADP to its peak amplitude **(B)**.

The properties of submucosal AH and myenteric AH neurons—those characterized by prolonged AHPs following their action potentials—and S neurons that exhibit smaller action potentials without an AHP and prominent fast excitatory synaptic potentials following stimulation of their inputs (Hirst et al., 1974; Bornstein et al., 1994) were examined at least 10 min after impalement. Excitability of neurons was established by injecting depolarizing current pulse steps, 500 ms in duration and in 50 pA increments over the range 50–350 pA, through the recording electrode. To standardize the basal conditions, the membrane potential of the neurons was held at approximately −50 mV by passing current through the recording electrode, if necessary, and was returned to this value in between each change in current pulse amplitude. The number and duration of firing of action potentials (APs) triggered by each pulse was determined. Duration of firing was measured as the time between the start of the first AP to then end of the last AP triggered by the pulse. When a single AP was observed, the duration was taken to be the time between the start and end of the AP. Neurons that failed to fire were treated as having a duration of 0 ms. Instantaneous firing frequency was measured as the interspike interval (ISI) between the first two spikes at the 300 pA current step. To examine the input resistance of neurons, hyperpolarizing current pulses (500 ms duration, 50 pA increments over 50–350 pA) were injected through the electrode. The voltage/current relationship at each pulse amplitude was plotted and the gradient of the curve was used to derive the input resistance of the cell (MΩ) according to Ohm's law (V = IR; voltage = current × resistance). *I*_h_ currents can be detected as a characteristic “sag” in measured membrane potential present during hyperpolarizing steps. The magnitude of the *I*_h_-induced rectification was measured as the difference between the maximum hyperpolarization and the hyperpolarization at the end of each current step (Figure 1). The gradient of this voltage current relationship was then determined to calculate the change in IR due to the conductance change underlying the *I*_h_ current, in accordance with Ohm's law.

A focal stimulating electrode was positioned on the mucosa opposite a fiber tract from a chosen ganglion or on an interganglionic fiber tract leading into that ganglion. Single stimuli were used to evoke individual antidromic action potentials in AH neurons. The amplitude (change in mV, from baseline to peak amplitude) and duration (D_50_) (time taken to return to half amplitude from peak amplitude) of the APs were measured. The amplitudes, D_50_s and full durations (time from the beginning of the AP to when membrane potential returned to baseline) of the characteristic AHPs of AH neurons were measured. In addition, the amplitudes of after-depolarizing potentials (ADP) were measured from the base of the ADP to its peak amplitude (Figure 1). Trains of stimuli (10 pulses, 20 Hz) were used to evoke slow excitatory postsynaptic potentials (sEPSPs). The amplitude and D_50_ of sEPSPs were measured.

### Immunohistochemistry

In order to identify neurons as either a subclass of motor or interneuron, after each experiment, the morphology of impaled neurons and, when possible, immunoreactivity for nNOS was examined. Preparations were fixed overnight in Zamboni's fixative (2% formaldehyde/0.2% picric acid in 0.1 M phosphate buffered saline, PBS, pH 7.0). The fixative was cleared with 3 × 10 min washes in dimethylsulfoxide (DMSO), followed by 3 × 10 min washes in phosphate buffered saline (PBS) and the mucosa was removed if present. A primary antibody (AB) against nNOS (sheep anti-nNOS, Emson) was applied and incubated for 2–3 nights. Excess primary AB was washed with PBS. A secondary AB to visualize nNOS expression (Donkey anti-sheep 488, Molecular Probes) was added with streptavidin anti-biotin alexa 594 (Molecular Probes) to allow identification of impaled neurons and incubated for 3 h in dark humid containers. Secondary ABs were removed by washing in PBS and the preparations were mounted on glass slides in DAKO mounting medium (Carpinteria, CA, USA). Preparations were visualized using 40x and 63x oil objectives on a BIO-Rad MRC 1,024 confocal microscope mounted on a Zeiss fluorescence microscope (Gladesville, NSW, Australia) and images of impaled neurons were taken as a z series using separate filters for green and red at a resolution of 512 × 512 pixels. Images were later merged (Image J® software) to identify impaled neurons as nNOS positive (^+^) or negative (^−^).

### Drugs and toxins

Drugs were diluted in distilled water to make stock solutions and then again in saline to the working concentration on the day of experimentation. Drugs used were hyoscine (1 μM), nicardipine (1.25 μM) (both from Sigma Aldrich), CT (12.5 μg/ml, List Biologicals, Campbell, CA), tetrodotoxin (TTX, 1 μM, Alomone Labs, Jerusalem, Israel), granisetron (1 μM) (Smith Kline Beecham, Harlow Essex, UK), neurokinin 1 (NK1) antagonist SR140333 (100 nM) and NK3 antagonist SR142801 (100 nM) (both kind gifts from Dr Emonds-Alt, Sanofi Recherche, Montpellier, France).

### Analysis and statistics

The electrophysiological data were analyzed using AxoScope computer software (version 10.2.0.14, Axon Instruments U.S.A.). The injection of depolarizing and hyperpolarizing current pulses into the neurons was performed twice per cell, while the single and train stimulus regime was repeated 3 times to obtain averages for these parameters. Data are presented as mean ± SEM where *n* = number of neurons.

In the text below, quantitative comparisons are provided for numbers of APs and duration of firing for 300 pA depolarizing pulses. In addition, the number of APs fired and the firing duration have been plotted against the entire current pulse amplitude range to produce stimulus-response curves for each condition. Here statistical comparisons include analysis of the firing over the whole range of current pulses using two way ANOVA, where statistical values are given for the entire curve. Tukey's *post-hoc* tests were used to identify differences for individual current pulse amplitudes. Any such differences are specified in the results. Where no specification exists, differences in firing are to be taken as occurring over the entire curve, rather than at individual data points. Statistical analyses were made using 2-sample *t*-tests for independent groups or two way ANOVA for the stimulus-response curves, with *p* < 0.05 considered statistically significant.

## Results

In this study, CT incubation produced enhanced secretion in all preparations as previously described (Gwynne et al., 2009). Increased fluid accumulation was observed in jejuna that were injected with CT compared to saline controls after 90 min.

**Figure 2:**
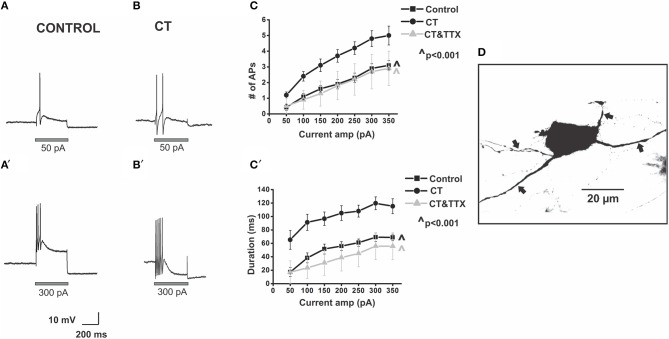
**CT increases the excitability of myenteric AH neurons close to the mucosa**. Example electrophysiological recordings of action potential firing evoked by current pulses of 50 pA (top panels) and at 300 pA (bottom panels) from a control **(A,A')** and CT (12.5 μg/ml) pre-treated **(B,B')** neuron. Graphical representations of the mean number **(C)** and total duration **(C**′**)** of APs fired at each depolarizing current amplitude (50–350 pA). Following CT treatment, AH neurons fired significantly more APs (^∧^*p* < 0.001) and for longer (^∧^*p* < 0.001) compared to neurons in control preparations. Firing was reduced following co-incubation of CT with TTX (1 μM), shown in grayscale (^∧^*p* < 0.001, firing and duration between CT and CT and TTX-treated preparations), returning to a control level (*p* > 0.05 control vs. CT and TTX preparations). A confocal micrograph of a myenteric AH neuron injected with biocytin during recording **(D)** arrows denote axons.

**Figure 3:**
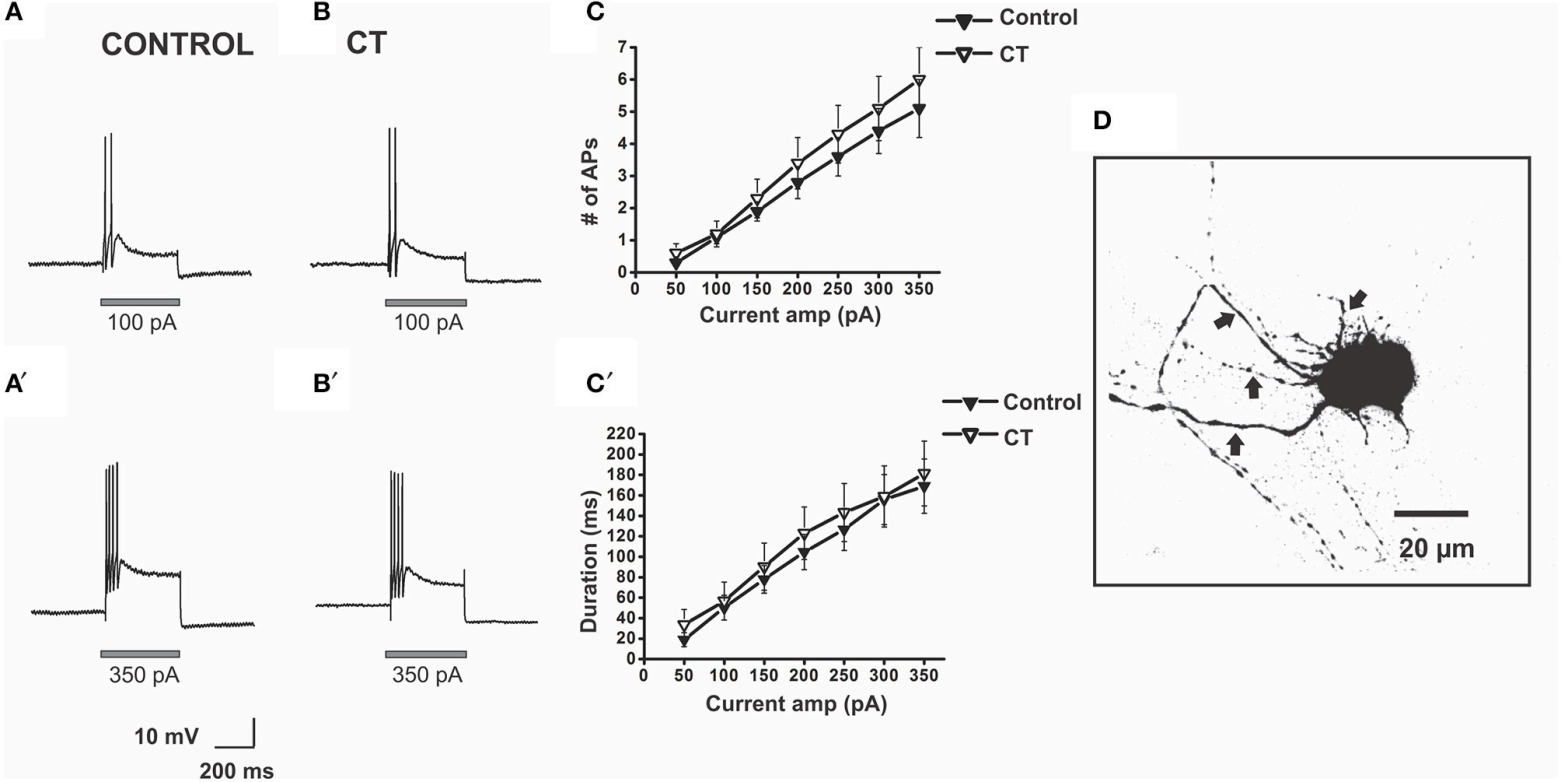
**CT did not affect the excitability of submucosal AH neurons close to the mucosa**. Example electrophysiological recordings of action potential firing at current pulses of 100 pA (top panels) and at 350 pA (bottom panels) from a control **(A,A')** and a CT (12.5 μg/ml) pre-treated **(B,B')** neuron. Graphical representations of the mean number **(C)** and total duration **(C**′**)** of APs fired at each depolarizing current amplitude (50–350 pA). The number of APs fired and their duration were not affected by CT pre-treatment. A confocal micrograph of an AH neuron in the SMP injected with biocytin during electrophysiological recording **(D)** arrows denote axons.

A total of 60 submucosal AH neurons and 111 myenteric AH neurons was examined electrophysiologically. All neurons displayed phasic firing properties (firing rapidly at the onset of the depolarization, then accommodating to the stimulus), regardless of treatment. The morphologies of 12 myenteric and 46 submucosal AH neurons were examined after electrophysiological experiments and processed to confirm and correlate their morphology with their electrophysiological properties. As expected all AH neurons had large cell bodies and displayed multiple axonal processes, S neurons typically had smaller cell bodies, short dendrites and a single axon (Bornstein et al., 1984, 1991a, 1994) (Figures 2D, 3D, **6D**).

### CT increases excitability of myenteric AH neurons and the effect is neurally mediated

We have previously shown that most submucosal S neurons exhibit an increase in excitability following CT treatment, but only when recorded close to intact mucosa (Gwynne et al., 2009). Thus, the firing properties of control and CT (12.5 μg/mL)-treated AH neurons in the first row of ganglia, close to the mucosa in mucosa-LMMP preparations were examined.

Myenteric AH neurons in CT-treated preparations (*n* = 29) were significantly more excitable than control neurons (*n* = 24). CT-treated neurons fired more action potentials (*p* < 0.001, at 200–350 pA, Tukey's *post-hoc* analysis) and for a longer duration (*p* < 0.001, at 50–350 pA, Tukey's *post-hoc* analysis) than controls [firing at 300 pA control 2.9 ± 0.3 APs over 69 ± 6 ms, CT 4.8 ± 0.5 APs over 120 ± 9 ms (Figure 2)], with firing more likely to occur at 50 pA following CT incubation (Control: 0.42 ± 0.2 APs; CT 1.2 ± 0.2 APs, *p* ≤ 0.05, 2-sample *t*-test) implying a lowered threshold for firing following CT pre-treatment. No change in the ISI was observed between the two groups (Control: 23.9 ± 1.3 ms; CT: 23.8 ± 1.5 ms, at 300 pA) indicating that their maximum firing frequency was unchanged at about 40 Hz, despite being more excitable.

The CT-induced increase in excitability was neurally mediated. Neurons incubated with CT together with TTX (1 μM) (*n* = 6) fired significantly fewer action potentials (*p* < 0.001) and had a shorter duration of firing (*p* < 0.001) than neurons incubated with CT alone [CT + TTX: 2.7 ± 1.1 APs at 300 pA, CT + TTX: 56 ± 20 ms at 300 pA (Figure 2)]. Firing was returned to a control level following co-incubation of CT with TTX (*p* > 0.05 between control and CT and TTX-treated preparations; Figure 2).

### Effect of CT on the membrane properties and synaptic potentials of myenteric AH neurons

To further examine this CT-induced excitability increase, a number of membrane properties and synaptic potentials of AH neurons were analyzed. The late AHP is critical in determining the excitability of AH neurons, since it has the capacity to limit both firing and slow excitatory transmission (Chambers et al., 2014). The amplitude of the AHP (*p* < 0.01, *n* = 17) and its duration (*p* < 0.05, *n* = 15) were significantly reduced following CT treatment (amplitude: control 6.5 ± 0.8 mV, CT 3.9 ± 0.5 mV; duration: control: 15.5 ± 2.6 s, CT 8.5 ± 2.0 s) (Table 1). While the exact physiological function of the ADP has not been determined, our computational model predicts that an increase in ADP magnitude can increase the excitability of AH neurons in some ways (Chambers et al., 2014). Pursuant to this, we found a significant increase in ADP amplitude observed in CT-treated neurons compared to neurons in the control group (*p* < 0.05, *n* = 17; control 8.5 ± 1.1 mV, CT 13 ± 1.3 mV) (Table 1).

**Table 1:** **Membrane properties and synaptic potentials of myenteric AH neurons close to mucosa following CT treatment**.

**Parameter**	**Control (n)**	**CT-treated (n)**
RMP (mV)	−55.4 ± 1(17)	−53.5 ± 1.1(24)
Input resistance (MΩ)	115.0 ± 6.8(22)	120.9 ± 6.5(22)
*I*_h_ current (MΩ)	25.9 ± 3.6(11)	27.2 ± 4.9(11)
AP amplitude (mV)	63.6 ± 3.0(14)	66.4 ± 2.5(17)
APD_50_(ms)	1.4 ± 0.1(14)	1.3 ± 0.1(17)
^*^AHP amplitude (mV)	6.45 ± 0.8(14)	3.9 ± 0.5(17) (*p* = 0.009)
^*^AHP full duration (s)	15.5 ± 2.6(13)	8.46 ± 2.0(15) (*p* = 0.036)
^*^ADP amplitude (mV)	8.5 ± 1.1(14)	13.0 ± 1.3(17) (*p* = 0.014)
Slow EPSP amplitude (mV)	8.5 ± 1.1(6)	7.4 ± 1.9(4)
Spontaneous AP firing %	8% (12)	8% (13)
Incidence Anode Break APs %	42% (12)	46% (13)

*The membrane and synaptic properties of neurons in the control group and CT- treated cells are compared*.

* *p < 0.05, n, sample size; RMP, resting membrane potential; IR, input resistance; AP, action potential; (I_h_), hyperpolarization-activated cation current; D_50_, half duration; AHP, after-hyperpolarizing potential; ADP, after-depolarizing potential; slow EPSP, slow excitatory post-synaptic potential. 2-sample t-test*.

CT pre-treatment did not alter the resting membrane potential, AP amplitude, AP duration (D_50_), input resistance or the *I*_h_ rectification in the myenteric AH neurons (Table 1). The amplitude and duration of sEPSPs that were evoked by trains of electrical stimuli were also unchanged by CT treatment. Similarly CT pre-treatment did not produce any changes in the incidence of spontaneous APs or anode break APs (Table 1).

### Firing of submucosal AH neurons is unaltered by CT

The firing properties of control and CT (12.5 μg/mL)-treated submucosal AH neurons in the first row of ganglia, next to the mucosa, in mucosa-SMP preparations were examined.

There was no significant difference between the stimulus-response curves for submucosal AH neurons in the CT-treated group (*n* = 33) and the equivalent curves recorded under control conditions (*n* = 27). The number of APs fired and the duration of firing evoked by the 300 pA current pulse stimulus were unchanged [Control 4.4 ± 0.7 APs; CT 5.1 ± 1.0 APs, Control: 156 ± 25 ms; CT 159 ± 30 ms, *p* > 0.05 (Figure 3)]. Furthermore, CT treatment did not affect input resistance, resting membrane potential and the amplitudes and durations of electrically evoked slow excitatory synaptic potentials (sEPSPs) of the recorded neurons (data not shown).

### Excitability of myenteric AH neurons is affected by proximity to intact mucosa

We tested whether the presence of, or proximity to, the mucosa affects the excitability of AH neurons in control and CT conditions, as we have reported previously for submucosal secretomotor neurons (Gwynne et al., 2009).

**Figure 4:**
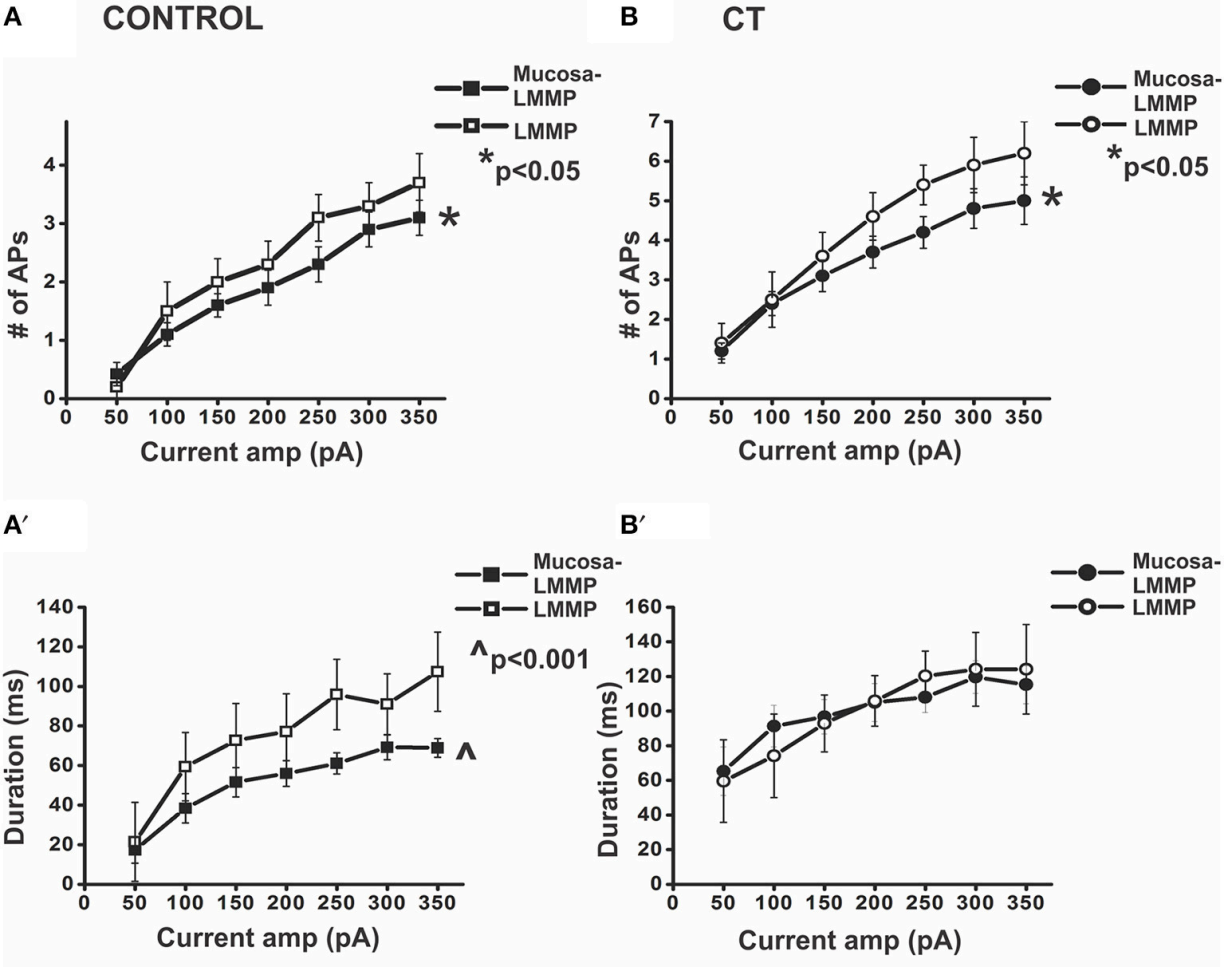
**Presence of the mucosa reduces action potential firing in myenteric AH neurons**. Graphical representations of the number of APs fired and the duration of firing during prolonged depolarization in myenteric AH neurons from the two control (preparations with and without mucosa) **(A,A′)** and two CT (12.5 μg/ml)-treated (preparations with and without mucosa) **(B,B′)** groups. In control conditions, neurons recorded from preparations without mucosa showed significantly higher number of APs fired **(A)** (^*^p < 0.05) and longer duration of firing **(A**′**)** (^∧^*p* < 0.001), compared to neurons located close to the mucosa. Following CT treatment, neurons from preparations devoid of mucosa displayed significantly higher number of APs fired **(B)** (^*^p < 0.05) compared to neurons located close to the mucosa. The duration of firing **(B**′**)** however, remained unaltered.

In contrast to submucosal secretomotor neurons (Gwynne et al., 2009), the presence of mucosa had an inhibitory effect on the excitability of myenteric AH neurons. In control conditions, AH neurons from stripped LMMP preparations (*n* = 13) fired more action potentials (*p* < 0.05) and for a longer duration throughout the pulses (*p* < 0.001) than AH neurons recorded in the first row of ganglia adjacent to the mucosa in mucosa-LMMP preparations (*n* = 24, Control 1st row 2.9 ± 0.3 APs; Control stripped 3.3 ± 0.4 APs, at 300 pA; Control 1st row 69 ± 6 ms, Control stripped: 91 ± 15 ms at 300 pA; Figure 4). In contrast, the ISI was greater in stripped preparations than adjacent to the mucosa (adjacent to mucosa 24 ± 1 ms, stripped: 30 ± 3 ms, at 300 pA, *p* < 0.05) indicating that the maximum instantaneous firing frequency was higher when the mucosa was present (42 Hz vs. 33 Hz respectively).

Similarly, in CT-treated preparations, myenteric AH neurons from fully stripped LMMP preparations (*n* = 5), exhibited more action potentials (*p* < 0.05) than those close to intact mucosa (*n* = 29, CT 1st row: 4.8 ± 0.5 APs; CT stripped: 5.9 ± 0.7 APs at 300 pA). However, there was no significant change in the duration of firing (CT 1st row: 120 ± 9 ms; CT stripped: 124 ± 21 ms at 300 pA, *p* > 0.9; Figure 4) or in the ISI (CT 1st row: 24 ± 2 ms; CT stripped: 19 ± 3 ms, at 300 pA, *p* > 0.05).

Proximity to the mucosa also affected the excitability of myenteric AH neurons. CT-treated myenteric AH neurons impaled further from the mucosa (*n* = 6) fired more APs (*p* < 0.001) and for longer (*p* < 0.05) than CT-treated neurons in the first row of ganglia (at 300 pA CT 1st row: 4.8 ± 0.5 APs; CT away: 6.5 ± 1.4 APs, CT 1st row: 120 ± 9 ms, CT away: 144 ± 31 ms). No difference in the ISI was observed between the two groups (CT 1st row: 24 ± 2 ms; CT away: 20 ± 1 ms, *p* > 0.1). Furthermore, CT-treated AH neurons from stripped preparations displayed firing (number of action potentials and duration of firing both *p* > 0.05) comparable to those from CT-treated preparations located away from the mucosa. Thus, all subsequent recordings were taken from preparations stripped of mucosa to further examine the components involved in sustained neuronal excitability.

### CT's effect on myenteric AH neurons is mediated by NK3, but not NK1 or 5-HT_3_ receptors

**Figure 5:**
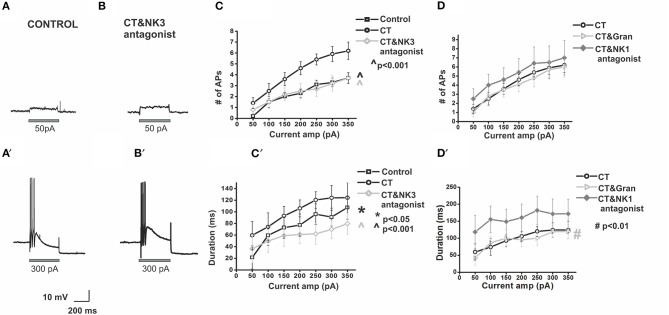
**Co-incubation of CT with a NK3 but not NK1 or 5-HT_3_ receptor antagonist prevents CT-induced hyperexcitability in myenteric AH neurons**. Electrophysiological recordings are displayed showing a control neuron **(A)** and a CT (12.5 μg/ml) + NK3 (100 nM) antagonist-treated neuron **(B)** at 50 pA **(A,B)** and 300 pA **(A′,B′)** current pulses. These neurons exhibit comparable action potential firing. Pre-treatment with the CT + NK3 antagonist significantly reduced the number of APs fired **(C)** (^∧^*p* < 0.001) during depolarization's and their firing duration **(C**′**)** (^∧^*p* < 0.001), over a range of current pulse amplitudes (effect shown graphically in grayscale). This effect was not observed following the CT + NK1 antagonist (100 nM) or CT + 5-HT_3_ antagonist (1 μM) co-incubation **(D,D′)**. Instead, duration of action potential firing was increased with the addition of the NK1 receptor antagonist (^#^*p* < 0.01) **(D**′**)**.

Since slow EPSPs in AH neurons are primarily mediated by tachykinins acting via NK1 and NK3 tachykinin receptors (Bertrand and Galligan, 1995; Alex et al., 2001; Johnson and Bornstein, 2004) and slow EPSPs suppress AHPs (Grafe et al., 1980) and therefore modify excitability of AH neurons, the effects of NK1 and NK3 antagonists on the CT-induced changes in firing properties of myenteric AH neurons were examined. The CT-induced increased firing in AH neurons was prevented by co-incubation with the NK3 receptor antagonist (SR142801, 100 nM). Following luminal co-incubation of CT with SR142801, myenteric AH neurons (*n* = 6) displayed a significant reduction in the number (*p* < 0.001) and duration of action potentials fired (*p* < 0.001) when compared with pre-incubation with CT alone [CT: 5.9 ± 0.7 APs; CT + NK3 antagonist: 3.2 ± 0.7 APs, CT: 124 ± 21 ms, CT + NK3 antagonist: 70 ± 16 ms, at 300 pA (Figure 5)]. Addition of the NK1 antagonist (SR140333, 100 nM), however, had no effect on the number of APs fired when compared with pre-incubation with CT alone (CT + NK1 antagonist: 6.5 ± 1.8 APs, at 300 pA, *n* = 6, *p* > 0.1). However, duration of action potential firing was increased (*p* < 0.01, at 300 pA, CT + NK1 antagonist: 172 ± 44 ms; Figure 5).

An established mechanism by which CT induces a massive secretion in the small intestine includes 5-HT release by enterochromaffin (EC) cells (Farthing, 2000) and 5-HT is known to excite AH neurons via 5-HT_3_ receptors on their mucosal terminals (Bertrand et al., 2000). Luminal co-incubation of CT with the 5-HT_3_ receptor antagonist granisetron (1 μM, *n* = 6), however, did not alter the number and duration of APs fired during the protocol, when compared with pre-incubation with CT alone [at 300 pA, CT + granisetron: 5.7 ± 0.9 APs, CT + granisetron: 119 ± 17 ms, *p* > 0.5 (Figure 5)].

### Myenteric S neurons show no change in firing following CT incubation

**Figure 6:**
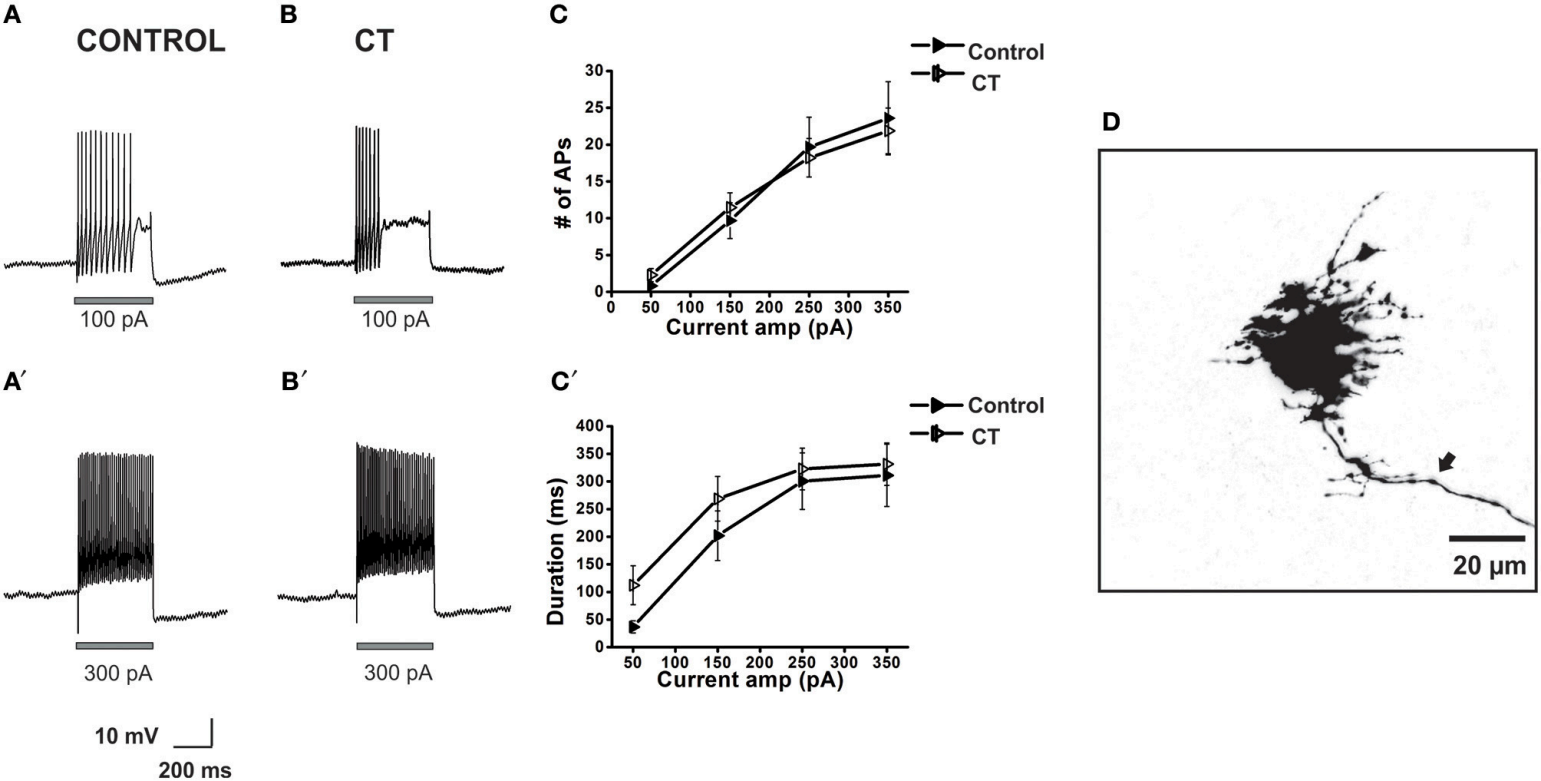
**Effects of CT on the action potential firing properties of myenteric S neurons close to the mucosa**. Example electrophysiological recordings of action potential firing at current pulses of 100 pA (top panels) and at 300 pA (bottom panels) from a control **(A,A')** and CT (12.5 μg/ml) pre-treated **(B,B')** neuron. Following CT treatment, firing number **(C)** of myenteric S neurons and firing duration **(C**′**)** was unchanged compared to neurons in control preparations. A confocal micrograph of a myenteric S neuron injected with biocytin during recording **(D)** arrow denotes axon.

Since myenteric AH neurons were hyperexcitable after CT incubation and are known to have synapses on almost all other types of myenteric neuron including interneurons and motor neurons which display S-type electrophysiology (Furness et al., 2004), we examined the excitability of 12 myenteric S neurons from control preparations and 24 S neurons from CT-treated preparations. No significant effects of CT incubation on excitability, resting membrane potential, input resistance, AP properties or spontaneous AP firing were identified (Figure 6, Table 2). A diverse range of S neurons exists that can be characterized by their morphology, axonal projections and immunoreactivity for nNOS. While we found no difference in excitability between control and CT-treated S neurons in the MP as a whole, it is likely that we have only sampled a small portion of the different subclasses of S neurons that exist and we cannot rule out effects in theses sub-populations. We also cannot exclude that these sites in the network respond to CT exposure with immediate effects during incubation. The morphology and axonal projections were examined in 11 control S neurons; 2 neurons projected orally, 1 projected circumferentially 8 had axons projecting anally. Immunoreactivity for nNOS was detected in 4 of these neurons (all projected anally and 2 were identified as inhibitory motor neurons with another being a descending interneuron and the 4th being unclassifiable). Axonal projections were examined in 21 (of 24) CT neurons; 4 projected orally, 4 projected circumferentially and 13 anally. Nineteen were classified further as interneurons (*N* = 10) or motor neurons (*N* = 9) and 5 (of 14) were NOS immunoreactive.

**Table 2:** **Membrane properties and synaptic potentials of myenteric S neurons in control and CT-treated preparations**.

**Parameter**	**Control (n)**	**CT-treated (n)**
RMP (mV)	−36.7 ± 4.6(9)	−37.4 ± 1.5(17)
Input Resistance (MΩ)	165.9 ± 17.8(8)	158.5 ± 7.0(15)
AP amplitude (mV)	52.2 ± 2.1(11)	55.2 ± 1.9(20)
APD_50_(ms)	0.80 ± 0.03(11)	0.78 ± 0.04(20)
Fast EPSP Amp (mV)	31.0 ± 2.4(11)	26.8 ± 1.5(19)

*n, sample size; RMP, resting membrane potential; AP, action potential; ADP, after-depolarizing potential; D_50_, half duration; Fast EPSP, fast excitatory postsynaptic potential. 2-sample t-test*.

## Discussion

**Figure 7:**
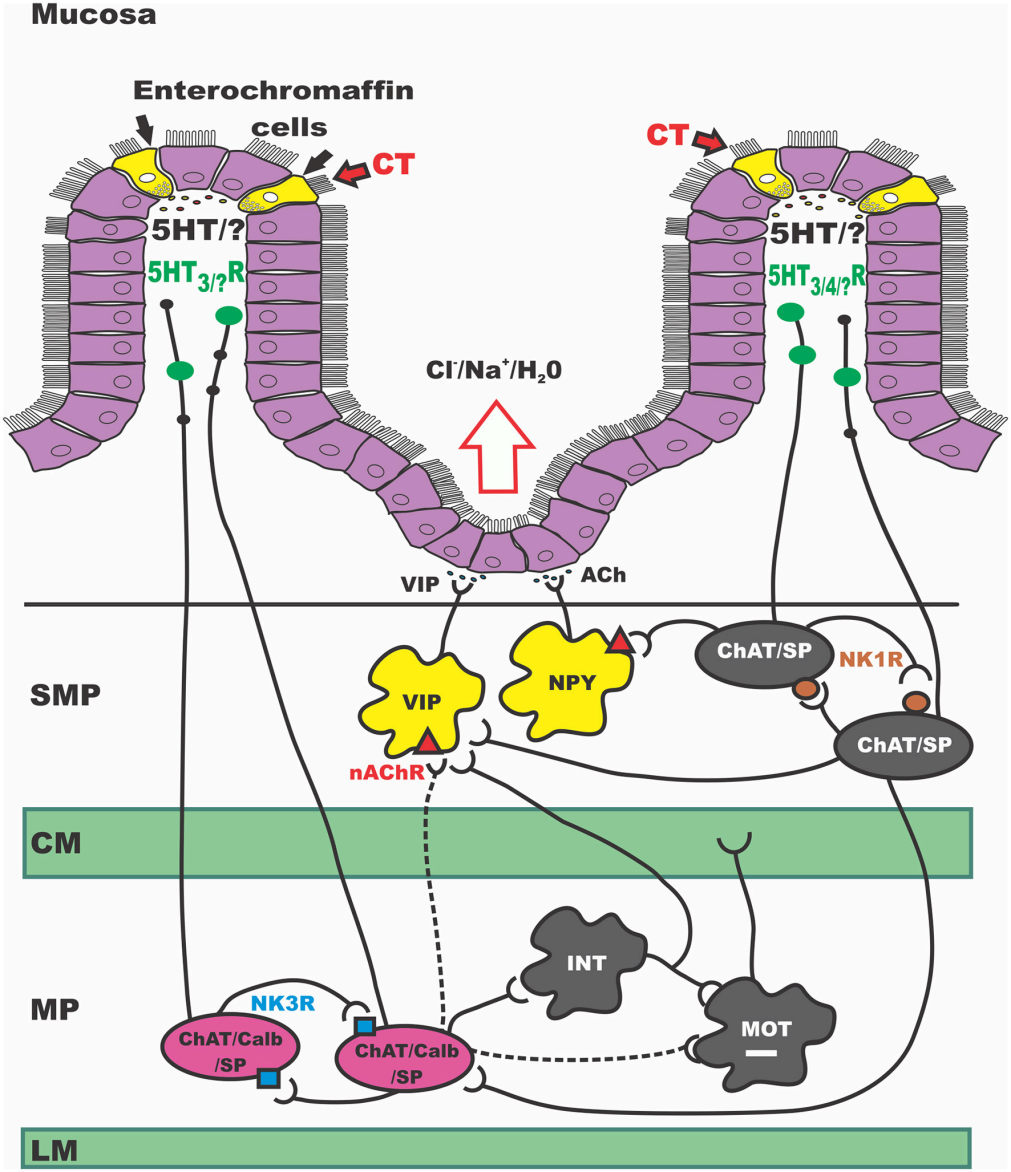
**Schematic of secretomotor pathways of the guineapig ENS activated by CT**. CT, Cholera toxin; SMP, submucosal plexus; CM, circular muscle; MP, myenteric plexus; LM, longitudinal muscle; VIP, vasoactive intestinal peptide; NPY, neuropeptide Y; Ach, acetylcholine; ChAT, choline acetyltransferase; nAChR, nicotinic acetylcholine receptor; SP, substance P; Calb, calbindin; INT, interneuron; MOT, motor neuron; NK1R, neurokinin 1 receptor; NK3R, neurokinin 3 receptor; 5HT, serotonin; 5HTR, serotonin receptor. This simplified schematic of the secretomotor circuit in the guinea pig small intestine integrates our current data with previous work and presents an updated model for CT-induced hypersecretion. It highlights several sites of sustained excitability following CT exposure. Firstly at the mucosal epithelium where CT produces a large release of 5-HT. In myenteric ISNs (in pink) and in secretomotor neurons in the SMP (in yellow). In this study, other myenteric and submucosal neuron subtypes did not show sustained changes in excitability; we cannot exclude however, acute effects occurring during CT incubation. Taken together the revised model proposes that CT acts at the mucosal epithelium to release a range of mediators from EC and EE cells. This CT exposure activates 5-HT-dependent and independent neural pathways: (1) a path where 5-HT acts on 5-HT_3_ receptors to inhibit motility, (2) a 5-HT -independent prolonged activation of myenteric ISNs (via NK3 receptors), possibly due to another mucosally-derived mediator, and (3) a 5-HT dependent pathway, presumably via 5-HT_3_ receptors on mucosal terminals of myenteric ISNs which leads to prolonged excitation of secretomotor neurons in the SMP.

The ENS has been implicated in CT-induced hypersecretion for over 30 years (Cassuto et al., 1981; Farthing, 2000; Lundgren, 2002; Burleigh and Banks, 2007), but the specific neurons affected and their roles in the enteric neural circuit have not been fully characterized. We have reported that incubation of the guinea pig jejunum with CT in the lumen *in vitro* induces a sustained increase in the excitability of submucosal secretomotor neurons (Gwynne et al., 2009). We have now examined the effects of CT on excitability of other functionally distinct subclasses of neurons and identified important changes in the firing properties of myenteric AH neurons. CT pre-treatment increased excitability of myenteric AH neurons, but not submucosal AH neurons or myenteric S neurons. This effect was dependent on neural activity and required activation of NK3 tachykinin receptors during the incubation period. Blocking NK1 or 5-HT_3_ receptors during the incubation did not alter the effect of CT. This suggests that CT produces hypersecretion via prolonged increases in the responsiveness of both the afferent and efferent limbs of secretomotor circuits (Figure 7).

### CT increases the excitability of myenteric AH neurons, but not submucosal AH neurons or myenteric S neurons

Prior incubation of CT in the lumen of isolated segments of guinea pig jejunum caused myenteric AH neurons to respond to smaller depolarizations and for longer, but the maximum firing frequencies remained constant. The increased excitability of myenteric AH neurons was not accompanied by increased spontaneous firing of these neurons or changes in slow EPSPs. Thus, the increased excitability is due to a change in the intrinsic properties of these AH neurons rather than a change in their inputs.

Submucosal AH neurons were unaffected by prior luminal incubation with CT. The large sample of recordings of submucosal neurons were made in ganglia adjacent to intact mucosa, where increased excitability of secretomotor neurons results from identical CT incubations (Gwynne et al., 2009). However, excitability of myenteric AH neurons was increased both in ganglia adjacent to intact mucosa and in fully stripped preparations. Thus, it is unlikely that the failure to detect a change in excitability was due to either the study being underpowered or the experimental conditions.

Myenteric AH neurons respond to mechanical deformation of the muscle (Kunze et al., 2000), muscle contractile activity (Kunze et al., 1999) and chemical stimuli applied to the mucosa (Kunze et al., 1995; Bertrand et al., 1997; Gwynne and Bornstein, 2007a), but not to mucosal deformation (Bertrand et al., 1997). In contrast, the very limited published work on sensory transduction by submucosal AH neurons indicates that they respond to mucosal deformation (Kirchgessner et al., 1992). Thus, our data suggest that CT preferentially enhances responses of enteric pathways to some sensory modalities, but not others.

Luminal CT excites propulsive intestinal motility (Fung et al., 2010) and triggers hypersecretion in the guinea pig small intestine (Carey and Cooke, 1986; Gwynne et al., 2009). The motility effects were observed when CT was present in the lumen (Fung et al., 2010), over an equivalent incubation period to the present study. Contractile responses of rat jejunum *in vivo* are enhanced after 120 min of CT incubation and persist for several hours after washout of the toxin (Kordasti et al., 2006). Thus, the excitability of myenteric interneurons and motor neurons might be affected by incubation with CT. These neurons are all classed electrophysiologically as S neurons. The number of functional subtypes of S neurons, however, is large and some sub-populations form as little as 1% of the total, although their outputs diverge to contact many myenteric and submucosal neurons (Pompolo and Furness, 1995; Moore and Vanner, 2000; Bornstein et al., 2004). Such neurons did not contribute significantly to our sample, as recordings from at least 1,000 neurons in each condition would be required. Thus, we cannot rule out significant effects on some myenteric S neurons, but our data indicate that most interneurons in pathways regulating secretion or motility and the output neurons of motility pathways are unaffected by CT.

### CT increases the excitability of myenteric AH neurons via a mechanism requiring neural activity

To test whether the increased excitability of myenteric AH neurons seen after CT incubation depended on neural activity during the incubation period, we blocked voltage-dependent sodium channels with TTX while CT was in the lumen. This prevented the CT-induced increased excitability of the AH neurons, as seen with submucosal secretomotor neurons (Gwynne et al., 2009). We have shown that CT does not penetrate the mucosa during this type of incubation, with staining for the CT-B subunit confined to the mucosa and not in the underlying SMP, MP or muscle (Gwynne et al., 2009). Thus, CT does not interact directly with enteric neurons, strongly implicating indirect activation of enteric neural pathways by luminal CT as the mechanism responsible for the enhanced excitability. It is unlikely that the failure of CT to enhance excitability is due to persistence of TTX beyond the incubation, because impalements were often 5–6 h after the toxins had been washed out and TTX is reversible in guinea pig myenteric plexus (Hirst et al., 1974).

It is also possible that activation of extrinsic primary afferent neurons with axon collaterals in the myenteric plexus contributes to induction of hyperexcitability in myenteric AH neurons. That such collateral innervation of myenteric plexus exists in guinea pig small intestine is indicated by the findings of Takaki and Nakayama (1990), who showed that stimulation of mesenteric nerves as they enter the ENS evokes sEPSPs in myenteric AH neurons. However, extrinsic primary afferents probably do not have a major role, because long-term extrinsic denervation of the rat small intestine does not prevent CT-induced hypersecretion *in vivo* (Sjöqvist, 1991; Turvill et al., 2000). Further, submucosal ganglia in guinea pig small intestine receive inputs from SP-containing extrinsic nerve fibers (Costa et al., 1981), but submucosal AH neurons are unaffected by CT.

### CT probably acts to reduce IK channel activity in AH myenteric neurons

Myenteric AH neurons express a wide variety of different ion channels that contribute to their excitability (Chambers et al., 2014). For example, inflammation-induced increased excitability of these neurons is associated with an increase in an hyperpolarization-activated cyclic nucleotide dependent cation conductance (*I*_*h*_*)*, (Linden et al., 2003) whose activation reduces the amplitude and duration of the slow AHP in these neurons (Chambers et al., 2014). In contrast, CT does not cause inflammation or alter *I*_*h*_, but does reduce both the amplitude and duration of the AHP in myenteric AH neurons (Table 1). AHPs in these neurons result from the opening of an intermediate conductance calcium-dependent potassium channel (IK) as a consequence of Ca^2+^ entry during the action potential (Hirst et al., 1974, 1985; Vogalis et al., 2002; Neylon et al., 2004). Realistic computational modeling shows that reducing IK conductance following an action potential increases numbers of action potentials fired and durations of firing evoked by depolarizing current pulses (Chambers et al., 2014). In the model, this occurs with little change in resting membrane potential, input resistance or the maximum frequency of firing, findings similar to our observations of the effects of CT pre-treatment. The ADP triggered by individual action potentials in AH neurons is enhanced by CT. However, our computational simulations indicate that changes in the ADP do not produce changes in action potential firing that match those seen with CT pre-treatment (Chambers et al., 2014) thus the function of this particular membrane event remains unclear.

The reduction in the AHP produced by CT may be due to an increased drive in a protein kinase pathway(s). IK channel phosphorylation by either protein kinase A (PKA) or protein kinase C (PKC) is a major source of potassium channel regulation in AH neurons (Del Carlo et al., 2003; Vogalis et al., 2003). The open probabilities of these channels are reduced by phosphorylation by PKA, which suppresses the AHP (Vogalis et al., 2003). As the CT effect depends on activation of NK3 tachykinin receptors (see below), which are coupled to phospholipase C, the PKC pathway may contribute to suppression of the AHP following CT treatment. PKD, a downstream target of PKC, is of particular interest. In guinea pig ileum, stimulating the NK3 receptor responsible for slow EPSPs in AH neurons, translocates PKD from the cytosol to the plasma membrane where it is subsequently phosphorylated (Poole et al., 2008). PKD activation may account for the membrane changes underlying CT-induced changes in AH neuron excitability.

### Role of mucosal mediators in CT-induced effects on enteric neurons

The current model of the mechanism underlying CT-induced hypersecretion can be summarized as CTB-subunits binding to GM-1 gangliosides in the apical membrane of enterochromaffin cells (Farthing, 2000; Burleigh and Banks, 2007). This is believed to result in depolarization, increased Ca^2+^ entry via voltage-dependent Ca^2+^ channels and release of serotonin from the basolateral surface. The serotonin is thought to activate 5-HT_3_ receptors on mucosal terminals of myenteric AH neurons (Bertrand et al., 2000), thereby activating secretomotor pathways.

We found that only secretomotor neurons close to intact mucosa exhibit increased excitability in CT pre-treated preparations (Gwynne et al., 2009). However, the enhanced excitability of myenteric AH neurons was seen in in preparations lacking any mucosa and thus could not be due to ongoing activity of CT in the mucosa. These findings highlight the conclusion that the prolonged effects of CT are not simply due to an irreversible action on mucosal epithelial cells, whether enterocytes or enterochromaffin cells, as has been widely postulated. Further, mucosal mediators may have different effects on the afferent and efferent limbs of the neurogenic pathway, or perhaps the reflex limbs are affected by different mucosal mediators.

Our data show that increased excitability of myenteric AH neurons does not depend on activation of 5-HT_3_ receptors during CT exposure as shown by insensitivity to granisetron during CT incubation. Several other lines of evidence indicate that, although CT-induced hypersecretion depends on 5-HT_3_ receptor activation, other mediator/receptor combinations are also activated by CT. For example, luminal CT increases propulsive motor patterns in guinea pig jejunum within minutes of exposure, an effect enhanced by blocking 5-HT_3_ receptors (Fung et al., 2010). In this case, luminal CT increases activity of 5-HT_3_ receptors, but the pathway excited inhibits propulsive motor activity. Other mediators released from the mucosa may also have a role in CT-induced effects as EC cells contain several other mediators (e.g., cholecystokinin, secretin, ATP) (Cooke et al., 2003; Engelstoft et al., 2013; Gribble and Reimann, 2016) and presumably the same release processes act on these along with serotonin. Further, it seems reasonable to assume mechanisms operating in enterocytes and EC cells also operate in enteroendocrine cells (Barber et al., 1986; Eklund et al., 1989; Lundgren, 1998; Gribble and Reimann, 2016). Future studies aimed at identifying mediators responsible for activation of this pathway will be important for understanding how CT affects the enteric neural circuitry.

### Role of tachykinin receptors

Myenteric AH neurons express both NK1 and NK3 tachykinin receptors. However, the effect of CT incubation on myenteric AH neurons depends on NK3, but not NK1, tachykinin receptors (Figure 5). This is consistent with earlier studies suggesting that these receptors mediate distinct membrane events (Johnson and Bornstein, 2004). NK3 receptors activate the Nav1.9 sodium channel via a PKC pathway (Copel et al., 2009). Computational simulations indicate that increasing Nav1.9 conductance increases both the number of action potentials and the duration of firing of AH neurons, but this is associated with an increased amplitude of the resulting AHP (Chambers et al., 2014). Whether activation of NK1 receptors modulates Nav1.9 in these neurons is unknown. Thus, whether the CT-induced increased excitability relates to the Nav1.9 activation by NK3 receptors requires further investigation.

Unlike myenteric AH neurons, submucosal AH neurons do not express NK3 receptors (Jenkinson et al., 1999), although they do express NK1 receptors (Portbury et al., 1996; Lomax et al., 1998; Harrington et al., 2005). It is tempting to suggest that the absence of NK3 receptors directly acting on the submucosal AH neurons accounts for the failure of CT incubation to enhance their excitability. However, VIP secretomotor neurons also rarely express NK3 receptors (Jenkinson et al., 1999), while the cholinergic NPY-containing and calretinin-containing secretomotor neurons do, and both VIP and NPY, but not calretinin, submucosal neurons are made hyperexcitable by CT incubation via an NK3 and NK1 receptor-dependent process. The nature of the second messenger systems activated by these two different tachykinin receptors in distinct classes of enteric neurons has not been identified. While both subtypes are usually thought to activate phospholipase C, NK1 receptors are known to activate two alternative pathways in other systems (Quartara and Maggi, 1997). These issues require further investigation.

### New proposed circuit

The data presented here suggest a new model for CT-induced neurogenic hypersecretion. This is illustrated in Figure 7, which shows the secretomotor circuit (simplified) and how luminal exposure of CT may produce hyperactivity at several sites. These include the intestinal epithelium itself, a major class of ISNs, myenteric AH neurons, and the output neurons of the secretomotor pathways with both cholinergic and non-cholinergic (VIP) secretomotor neurons being affected.

We postulate that CT acts to release several mediators from EC (and EE) cells in the mucosa including serotonin which activate underlying neural pathways. Initial exposure to CT activates at least three neural pathways, one of which involves serotonin acting on 5-HT_3_ receptors to inhibit motility, perhaps via the 5-HT_3_ dependent local inhibitory reflex pathway described by Gwynne and Bornstein (2007b). This immediate effect may be unrelated to the persistent hypersecretion or prolonged motility increases seen after CT exposure *in vivo* (Mathias et al., 1976).

The second pathway, highlighted in this present study, probably involves a mediator other than serotonin, does not appear to involve 5-HT_3_ receptors, and produces prolonged activation of NK3 tachykinin receptors on myenteric AH neurons. This may well be due simply to increased activity of these neurons during the incubation period, as they form recurrent excitatory networks with each other and communicate via NK3 receptor-mediated slow EPSPs (Bertrand and Galligan, 1995; Alex et al., 2001; Thomas and Bornstein, 2003; Johnson and Bornstein, 2004). Modeling has shown that increasing the excitability of the AH neuron network by reducing IK channel activity increases the gain in the output of the circuit, thereby amplifying sensory input into enteric neural pathways (Thomas et al., 2000; Thomas and Bornstein, 2003) and potentially leading to irreversible increases in network firing (Chambers et al., 2005). These neurons also have excitatory outputs to, and receive similar inputs from, the submucosal ISNs, (Galligan et al., 1988; Kirchgessner et al., 1992; Vanner and Macnaughton, 2004; Gwynne and Bornstein, 2007b) thereby adding a further level of amplification to the intrinsic sensory network.

The final pathway involves activation of 5-HT_3_ receptors, presumably on the mucosal terminals of ISNs, and leads to long lasting increases in excitability of the secretomotor neurons that innervate secretory enterocytes in the crypt regions. These increases in excitability involve both cholinergic and non-cholinergic secretomotor neurons and depend on proximity to the mucosa (Gwynne et al., 2009). The effect would be to amplify any activity in the secretomotor circuits arising from ongoing sensory stimulation or CT-stimulated release of mucosal mediators like serotonin.

Thus, we postulate that CT induces hypersecretion in the small intestine by indirectly increasing the excitability of both the input and the output arm of the secretomotor reflex pathway. This in turn is amplified by increased excitability of secretomotor neurons leading to increased secretion and potentially distension of the intestinal segment, thereby further exciting ISNs. Thus, we suggest that CT produces hypersecretion via a form of neural plasticity operating at several levels within the ENS.

## Conclusions and future directions

In light of the present study and previously published data (Gwynne et al., 2009), we propose a schematic diagram of the enteric circuitry involved in hypersecretory and propulsive effects of acute exposure to CT in the guinea pig small intestine (Figure 7). Prior exposure to CT leads to sustained excitability of specific groups of neurons in both enteric plexuses with the afferent arm residing in the myenteric plexus and efferent arm in the submucosal plexus. However, we cannot exclude the possibility that some enteric neurons are only activated during the incubation phase. The mechanisms involved in the increased excitability of these neurons and the involvement of substances other than 5-HT from the mucosa needs to be further investigated.

## Ethics statement

The study was carried out in accordance with the recommendations of the guidelines of the University of Melbourne Animal Experimentation Ethics Committee. The protocol was approved by the University of Melbourne Animal Experimentation Ethics Committee.

## Author contributions

KK undertook recordings from myenteric neurons, analyzed data and wrote the manuscript draft, RG undertook recordings from myenteric and submucosal neurons, analyzed data and helped revise manuscript, JF assisted with experimental design and figure construction, also revised manuscript, JB designed experiments, revised manuscript, obtained funds. All authors approved the final manuscript.

### Conflict of interest statement

The authors declare that the research was conducted in the absence of any commercial or financial relationships that could be construed as a potential conflict of interest.
